# Identification and Differentiation of Pseudomonas Species in Field Samples Using an *rpoD* Amplicon Sequencing Methodology

**DOI:** 10.1128/mSystems.00704-21

**Published:** 2021-08-03

**Authors:** Jonas Greve Lauritsen, Morten Lindqvist Hansen, Pernille Kjersgaard Bech, Lars Jelsbak, Lone Gram, Mikael Lenz Strube

**Affiliations:** a Department of Biotechnology and Biomedicine, Technical University of Denmark, Kongens Lyngby, Denmark; Pacific Northwest National Laboratory

**Keywords:** microbiome analyses, *Pseudomonas*, diversity, *rpoD*, 16S rRNA, amplicon sequencing

## Abstract

Species of the genus Pseudomonas are used for several biotechnological purposes, including plant biocontrol and bioremediation. To exploit the Pseudomonas genus in environmental, agricultural, or industrial settings, the organisms must be profiled at the species level as their bioactivity potential differs markedly between species. Standard 16S rRNA gene amplicon profiling does not allow for accurate species differentiation. Thus, the purpose of this study was to develop an amplicon-based high-resolution method targeting a 760-nucleotide (nt) region of the *rpoD* gene enabling taxonomic differentiation of Pseudomonas species in soil samples. The method was benchmarked on a 16-member Pseudomonas species mock community. All 16 species were correctly and semiquantitatively identified using *rpoD* gene amplicons, whereas 16S rRNA V3-V4 amplicon sequencing only correctly identified one species. We analyzed the Pseudomonas profiles in 13 soil samples in northern Zealand, Denmark, where samples were collected from grassland (3 samples) and agriculture soil (10 samples). Pseudomonas species represented up to 0.7% of the 16S rRNA gene abundance, of which each sampling site contained a unique Pseudomonas composition. Thirty culturable Pseudomonas strains were isolated from each grassland site and 10 from each agriculture site and identified by Sanger sequencing of the *rpoD* gene. In all cases, the *rpoD* amplicon approach identified more species than were found by cultivation, including hard-to-culture nonfluorescent pseudomonads, as well as more than were found by 16S rRNA V3-V4 amplicon sequencing. Thus, *rpoD* profiling can be used for species profiling of Pseudomonas, and large-scale prospecting of bioactive Pseudomonas may be guided by initial screening using this method.

**IMPORTANCE** A high-throughput sequencing-based method for profiling of Pseudomonas species in soil microbiomes was developed and identified more species than 16S rRNA gene sequencing or cultivation. Pseudomonas species are used as biocontrol organisms and plant growth-promoting agents, and the method will allow tracing of specific species of Pseudomonas as well as enable screening of environmental samples for further isolation and exploitation.

## INTRODUCTION

Pseudomonas species are ubiquitous and can be isolated from a range of environments, including plant rhizospheres, marine habitats, and animal tissues (1–4). While the genus contains species that are pathogenic to plants and animals, several species express traits that enable their use in bioremediation, plant growth promotion, or plant disease suppression (5–8). The underlying beneficial mechanisms are often linked to specific species or even strains, including the production of pathogen-suppressing secondary metabolites, such as the antibiotic 2,4-diacetylphloroglucinol (DAPG) and pyoverdine siderophores (5, 9–11). Also, some strains promote growth of plants by solubilizing inorganic nutrients, such as phosphate and iron, or by producing plant hormones (11–14). Members of the Pseudomonas fluorescens group, in particular, are a major source of bioactivity since they have markedly larger genomes than other pseudomonads (15) and a high number of biosynthetic gene clusters, as determined by genomic analysis (16). In addition to strains expressing these and other beneficial traits, it is also becoming clear that the structure and diversity of the Pseudomonas community in bulk and rhizosphere soils *per se* can be associated with suppression of crop fungal pathogens (17, 18). Studies on the distribution, abundance, and diversity of Pseudomonas spp. in soil and rhizosphere often rely on cultivation-dependent analyses. However, Aagot et al. and others have demonstrated that cultivation of individual species of Pseudomonas is dependent on the specific conditions used (e.g., level of nutrients), and the decision to use a specific cultivation medium is thus a source of bias (19). Given these biases, linking specific Pseudomonas species and/or community structures to certain ecosystem performance metrics (including suppression of crop fungal pathogens) remains a challenge.

Amplicon sequencing of the 16S rRNA gene has become the standard for culture-independent, taxonomic profiling of environmental microbial communities. However, the 16S rRNA genes are very similar across closely related Pseudomonas species, with less than 1% nucleotide dissimilarity between many of the species (20). For example, in the subgroup of P. putida, the dissimilarities are between 0.16 and 2.31% (20). Therefore, 16S rRNA gene profiling only provides taxonomic resolution at the genus level, and studies of Pseudomonas community structures and dynamics at the species level require sequencing and analyses of other housekeeping genes. The *rpoD* gene, which encodes the sigma 70 factor of RNA polymerase, is an excellent target gene for phylogenetic and taxonomic analyses of Pseudomonas species (21). Using a highly selective pair of Pseudomonas *rpoD* primers, PsEG30F and PsEG790R (21), an *rpoD* amplicon sequencing method was used to analyze environmental DNA obtained from a single water sample (22). The method was developed for the 454/Roche GS-FLX platform and used an in-house *rpoD* database for sequence analysis. Given the discontinuation of the 454/Roche GS-FLX platform and the understanding of Pseudomonas phylogeny, there is a need for development of an amplicon-based method for reliable identification and differentiation of Pseudomonas species from environmental samples.

The purpose of the present study was to develop an amplicon sequencing protocol compatible with the Illumina MiSeq 300PE platform and to establish a new and improved bioinformatic pipeline with an updated database built upon the Pseudomonas type strain collection and taxonomic framework from Hesse et al. (15). The *rpoD* amplicon method allowed Pseudomonas species differentiation in environmental soil samples and can guide future bioprospecting endeavors.

## RESULTS

### *In silico* target gene evaluation.

We evaluated nine genes and their accompanying primer sets (14 in total) for their phylogenetic discriminatory power using *in silico* PCR against two sets of genomes. The first was a library of the 166 type strain genomes of Hesse et al. (15) acting as a well-curated collection of all known Pseudomonas species and their phylogeny, although with most genomes being in contig form. The second was a library of 465 genomes of Pseudomonas species available from NCBI, all of which are complete but with high redundancy and incomplete taxonomy (Table 1). The *rpoD* primer pair PsEG30F and PsEG790R (21) resulted in the best phylogenetic resolution along with the highest total number of individual Pseudomonas genomes amplified and the lowest total non-Pseudomonas amplifications. This pair amplified 160/166 (96.39%) of type strains and 460/465 (98.92%) of the complete genomes (see Table S3 in the supplemental material), with no amplification of the negative controls. The *gyrB* gene primers UP-1E and APrU showed 100% amplification of the type strains, but unfortunately also amplified 25% of the negative controls and had multiple instances of amplicons much longer than the expected length. To further evaluate the phylogenetic resolution of the primers, a multiple-sequence alignment of the amplicons and the resulting phylogeny was compared to the study by Hesse et al. (15), in which the 166 distinct Pseudomonas type strains were phylogenetically resolved based on multiple-locus sequencing typing of 100 genes. Here, the *rpoD* primers PsEG30F and PsEG790R from Mulet et al. (21) produced amplicons with the closest similarity to the phylogenetic map of Hesse et al. (15) and separated all species in the phylogenetic tree. The primers generated an ∼760-nucleotide (nt)-long amplicon of the *rpoD* gene, which unfortunately led to a sequencing gap of 160 nt using the 300PE platform. Therefore, two new reverse primers were designed, PsJL490R and PsJL628R: however, both had lower amplification of the type strain genomes, at 89.16 and 83.73%, respectively. Moreover, the PsJL490R primer had poor *in vitro* amplification for the species of the synthetic community, while the PsJL628R primer showed unspecific amplification of the negative controls Stenotrophomonas maltophilia, Achromobacter xylosoxidans, and Azospirillum brasilense (see Fig. S1 in the supplemental material). Phylogenetic trees generated from these amplicons had comparable resolving power to the PsEG30F and PsEG790R pair, albeit with fewer nodes overall. As a consequence, we chose the PsEG30F and PsEG790R primer pair for Pseudomonas profiling. The universal 16S rRNA V3-V4 primers amplified 100% of the whole genomes and 87.95% of the type strains (owing to 16S genes often being the breakpoint in contigs). As noted previously (20), many of the amplicons are identical across Pseudomonas species and hence cannot be used for species resolution (see Fig. S2 in the supplemental material).

**TABLE 1:** Overview of genes and primers selected as primary targets for evaluation of 465 Pseudomonas strains with the *in_silico*_PCR algorithm

Target gene	Primer name	Sequence (5′→3′)	Length (nt)	Reference
16S rRNA	16S-341F	CCTACGGGNGGCWGCAG	464	42
	16S-805R	GACTACHVGGGTATCTAATCC		42
	16sF-LYP-3	GCGTAGAGTTTGATCCTGGCTCAG	1,253	26
	16sR-LYP-3	GACGGGCGGTGTGTRCA		26
	16S-rRNA-F	AGCGGCGGACGGGTGAGTAATG	1,300	27
	16S-rRNA-R	AAGGAGGGGATCCAGCCGCA		27
*atpD*	atpD-F	CTGGGCCGSATCATGGACG	900	27
	atpD-F	GTCCATGCCCAGGATSGCG		27
*carA*	carA-F	TTCAACACCGCCATGACCGG	700	27
	carA-R	TGATGRCCSAGGCAGATRCC		27
*gapA*	gapA-Fps	CGCCATYCGCAACCCG	690	28
	gapA-Rps	CCCAYTCGTTGTCGTACCA		28
*gltA*	gltA-F	GGTGACAATGGCATTCTGC	294	26
	gltA-R	GTGCTGCGGRTTATTGATGT		26
*gyrB*	gyrBBAUP2	GCGGAAGCGGCCNGSNATGTA		29
	APrU	GCNGGRTCYTTYTCYTGRCA		30
	UP-1E	AYGSNGGNGGNARTTYRA	888–891	30
	APrU	GCNGGRTCYTTYTCYTGRCA		30
*recA*	recA-F	TCSGGYAARACCACSCTGAC	600	27
	recA-R	RTACCAGGCRCCGGACTTCT		27
*rpoB*	LAPS	TGGCCGAGAACCAGTTCCGCGT	1,247	31
	LAPS27	CGGCTTCGTCCAGCTTGTTCAG		31
*rpoD*	PsEG30F	ATYGAAATCGCCAARCG	437	21
	PsJL490R	AGYTTGATYGGGATGAA		This study
	PsEG30F	ATYGAAATCGCCAARCG	575	21
	PsJL628R	GGGAACWKGCGCAGGAARTC		This study
	PsEG30F^*a*^	ATYGAAATCGCCAARCG	736	21
	PsEG790R^*a*^	CGGTTGATKTCCTTGA		21

a Primers used for this study.

10.1128/mSystems.00704-21.1 FIG S1 Gel electrophoresis of PCR products generated from members of the synthetic community using primer pairs (A) PsEG30F and PsEG790R, (B) PsEG30F and PsJL490R, and (C) PsEG30F and PsJL628R. Contrast has been enhanced. The size markers are 50-bp GeneRuler ladders (Thermo Fisher Scientific, Vilnius, Lithuania). Download FIG S1, PDF file, 0.1 MB. Copyright © 2021 Lauritsen et al. 2021 Lauritsen et al. https://creativecommons.org/licenses/by/4.0/ This content is distributed under the terms of the Creative Commons Attribution 4.0 International license.

10.1128/mSystems.00704-21.2 FIG S2 Phylogenetic tree of the *in silico* PCR amplicons generated from the 16S rRNA gene using V3-V4 primers. The PCR products were clustered at 97% similarity and aligned with MUSCLE v3.8.1551 (40), and a phylogenetic tree was generated with FastTree v2.1.10 (41) from the alignment. Tips are colored as clades generated by TreeCluster, with similar colors denoting highly similar or identical amplicons. Download FIG S2, PDF file, 1.2 MB. Copyright © 2021 Lauritsen et al. 2021 Lauritsen et al. https://creativecommons.org/licenses/by/4.0/ This content is distributed under the terms of the Creative Commons Attribution 4.0 International license.

10.1128/mSystems.00704-21.8 TABLE S3 *In silico* PCR amplification of 465 complete Pseudomonas genomes and 24 non-Pseudomonas genomes using 14 different primer pairs. Download Table S3, DOCX file, 0.01 MB. Copyright © 2021 Lauritsen et al. 2021 Lauritsen et al. https://creativecommons.org/licenses/by/4.0/ This content is distributed under the terms of the Creative Commons Attribution 4.0 International license.

### Synthetic community primer testing.

To benchmark the amplicon protocol, a mixture of DNAs from 16 Pseudomonas strains was used to test the performance of the candidate *rpoD* primers in comparison with the standard V3-V4 16S rRNA gene amplicon sequencing approach. The 16 strains were selected to challenge the method across the genus by including five groups of Pseudomonas species (P. aeruginosa, P. fluorescens, P. putida, P. stutzeri, and P. syringae) and on fine resolution by selecting closely related species especially within subgroups (P. fluorescens, *P. mandelii*, and *P. jessenii*). In contrast to the V3-V4 amplicons, PE300 Illumina reads of the *rpoD* amplicons do not overlap and hence cannot be analyzed by standard operational taxonomic unit (OTU)-based methods. To overcome this challenge, each read pair was instead aligned to a custom database of *rpoD* genes using bowtie2 (23).

**FIG 1:**
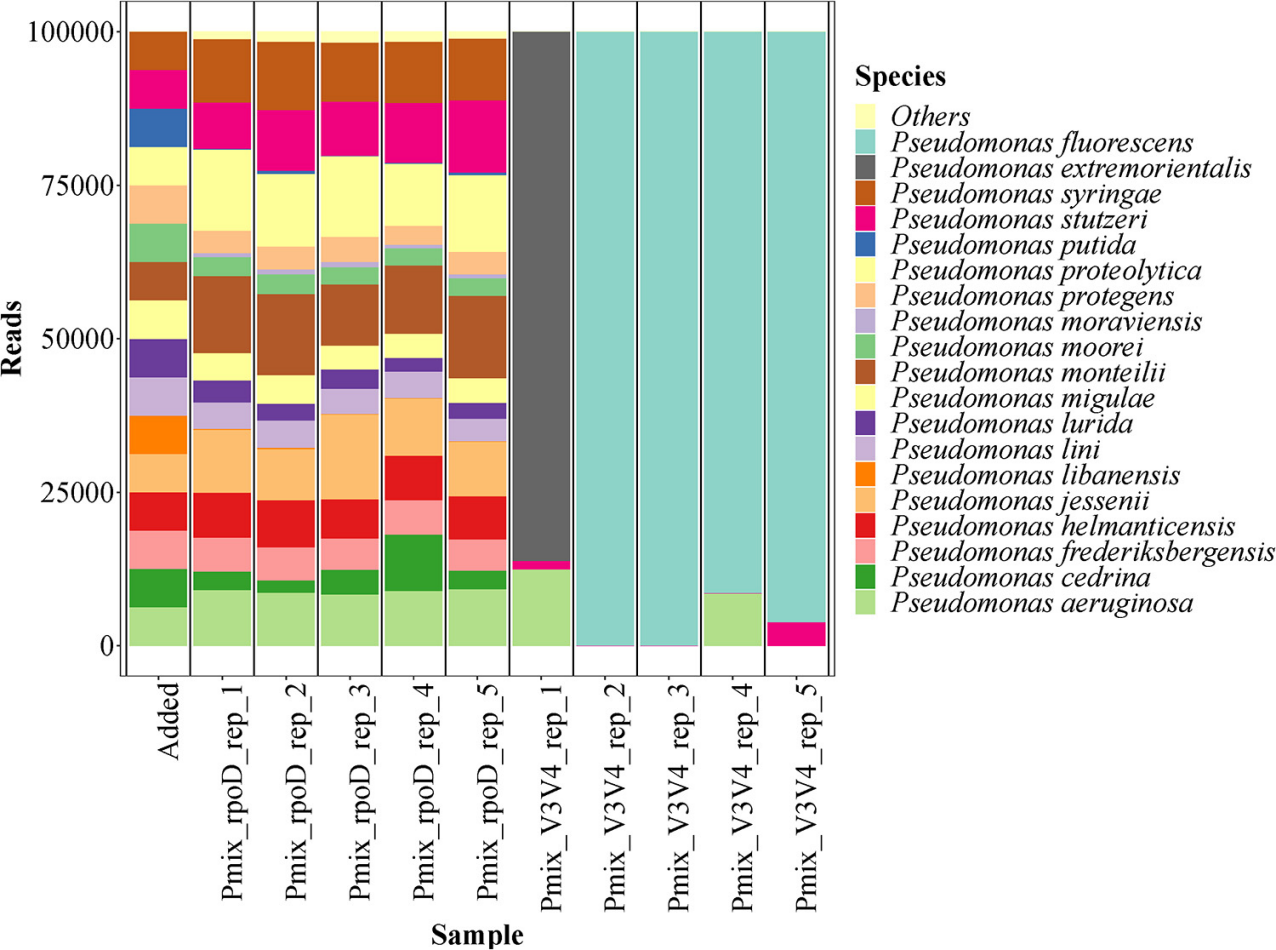
Composition of a defined mixture of DNAs from 16 Pseudomonas species as analyzed by *rpoD* gene amplicon sequencing and V3-V4 16S rRNA gene sequencing in comparison to the theoretical composition. The leftmost bar shows the assumed theoretical abundances in the defined Pseudomonas DNA mixture. Each sample has been normalized to 100,000 reads.

The *rpoD* amplicon method was able to identify all 16 strains, with abundances close to expected values (Fig. 1) and with a very low level of variation across the five technical replicates (Table 2). Of note, two species were underestimated: P. putida somewhat (∼4% of expected value) and *P. libanensis* severely so (∼1% of expected value). In contrast, the V3-V4 method erroneously classified the sample composition as being mainly P. fluorescens or *P. extremorientalis*, where the latter was not a part of the mixture. Also, small numbers of P. stutzeri and P. aeruginosa were detected by the V3-V4 approach. The beta dispersions—a measure of multivariate variation within groups—of the V3-V4 replicates were 4 times higher than those of the *rpoD* replicates, although this difference was not significant. The negative controls for both primer sets had low numbers of reads, later revealed to be common contaminants and adaptors.

**TABLE 2:** List of Pseudomonas species used in the artificial DNA mixture for positive control

Pseudomonas species	Strain	Group^*a*^	Type strain	*rpoD* relative ratio (%)^*b*^
P. aeruginosa	PAO1	P. aeruginosa	Yes	140.38 ± 5.59
*P. cedrina*	May11.4	P. fluorescens	No	69.38 ± 45.31
*P. frederiksbergensis*	Nina6.10	*P. mandelii*	No	85.08 ± 4.04
*P. helmanticensis*	Nina1.7	*P. koreensis*	No	114.60 ± 7.36
*P. jessenii*	May3.1	*P. jessenii*	No	161.69 ± 34.83
*P. libanensis*	Nina5.10	P. fluorescens	No	1.82 ± 1.00
*P. lini*	Nina1.6	*P. mandelii*	No	66.73 ± 4.50
*P. lurida*	Nina3.4	P. fluorescens	No	45.56 ± 8.51
*P. migulae*	DSM 17966	*P. mandelii*	Yes	67.20 ± 5.35
*P. monteil*	DSM 14164	P. putida	Yes	192.34 ± 23.44
*P. moorei*	DSM 12647	*P. jessenii*	Yes	47.53 ± 3.51
P. protegens	DTU9.1	P. protegens	No	58.01 ± 6.16
*P. proteolytica*	May3.3	*P. gessardii*	No	194.56 ± 20.10
P. putida	KT2440	P. putida	Yes	4.12 ± 2.71
P. stutzeri	DSM 5190	P. stutzeri	Yes	151.88 ± 24.48
P. syringae	DSM 10604	P. syringae	Yes	164.45 ± 9.25

a The groups and subgroups of Pseudomonas according to Hesse et al. (15).

b Observed values for *rpoD* relative abundance versus theoretical abundance are given as mean ± standard deviation, where 100% is the expected value.

### The microbial and Pseudomonas species composition in soil.

Soil was sampled from different sites, ranging from grassland to agricultural field soil. A total of 13 soil samples with 5 replicates each (65 samples in total) were analyzed, and after demultiplexing according to barcodes and primers, a total of 10,446,888 reads were available. Following this, 100,814 *rpoD* read pairs (201,628 reads) were annotated at the species level for the genus of Pseudomonas with sufficiently high confidence (minimum bit score of 10). According to the general purpose metagenomics classification tool Centrifuge (24), the non-Pseudomonas reads were approximately 50% PCR/adaptor artifacts (“Synthetic constructs”), along with the commonly found contaminant *Bradyrhizobium*, which was also found in the negative control. The overall mean and median values observed for the annotated *rpoD* reads across all samples were 1,558 and 598, respectively. The average number of Pseudomonas *rpoD* reads per sampling site varied between 133.4 (P8) and 4,022.2 (S7). Rarefaction curves revealed uneven saturation in some samples with low read depth (see Fig. S3 in the supplemental material), Moreover, we observed that quite a few read pairs were concordantly mapped, at 5.52%, likely owing to the high level of PCR artifact nonpseudomonal reads. In addition, less than 0.01% were discordantly mapped.

10.1128/mSystems.00704-21.3 FIG S3 Alpha diversity of the Pseudomonas population as described using the *rpoD* methodology. (Left) Rarefaction curves. (Right) Chao1 diversity. Download FIG S3, PDF file, 0.2 MB. Copyright © 2021 Lauritsen et al. 2021 Lauritsen et al. https://creativecommons.org/licenses/by/4.0/ This content is distributed under the terms of the Creative Commons Attribution 4.0 International license.

**FIG 2:**
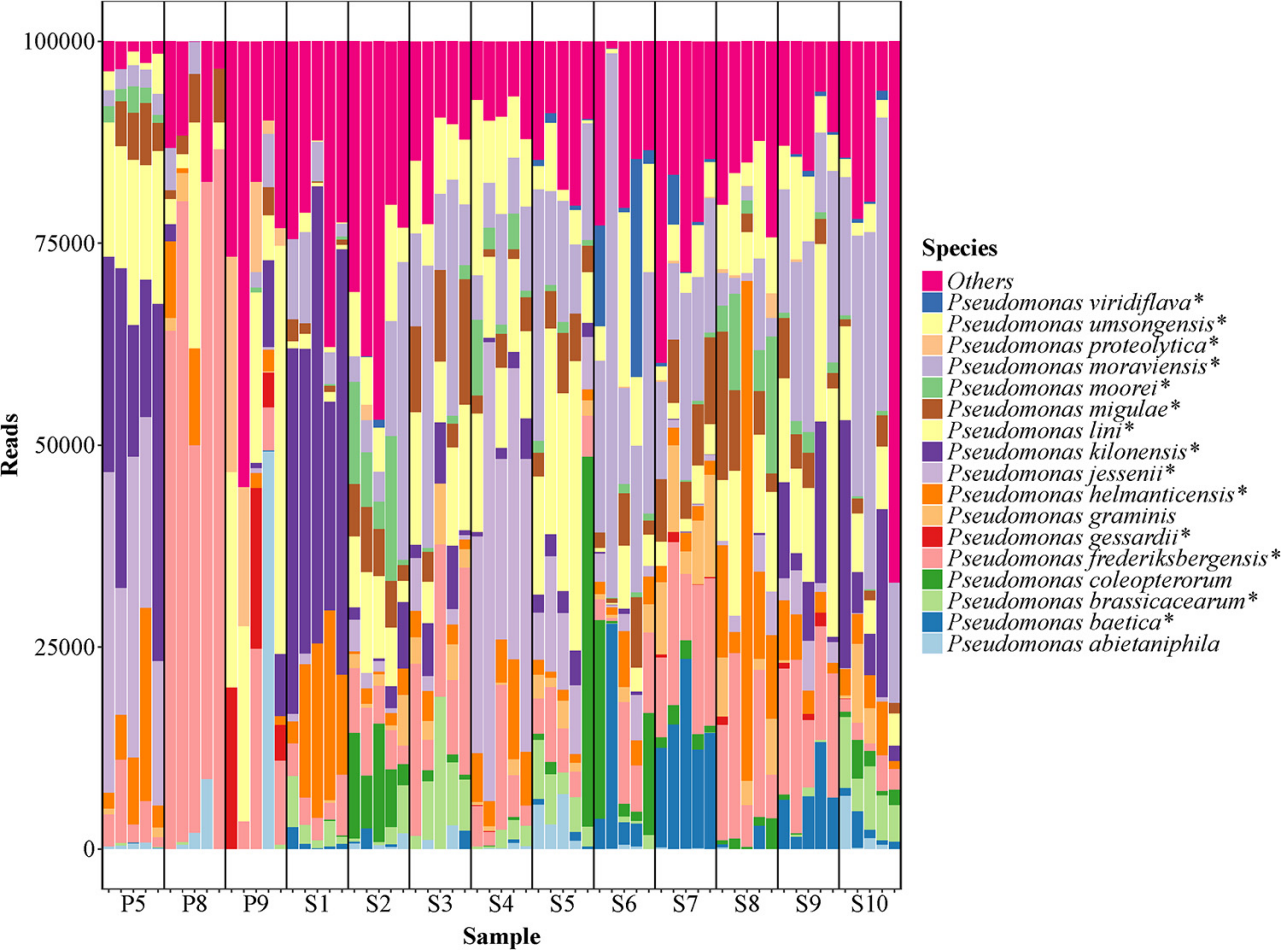
Relative abundances of the 20 most abundant Pseudomonas species in 13 soil samples as analyzed by *rpoD* amplicon sequencing. Each sample has been normalized to 100,000 reads. Fluorescent species are marked by *. S1, corn; S2, fallow (grass); S3, S7, and S9, wheat; S4, rye; S5, barley; S6, rapeseed; S8, grass seed; S10, Lucerne; P5 and P9, pristine short grass; P8, pristine long grass.

**FIG 3:**
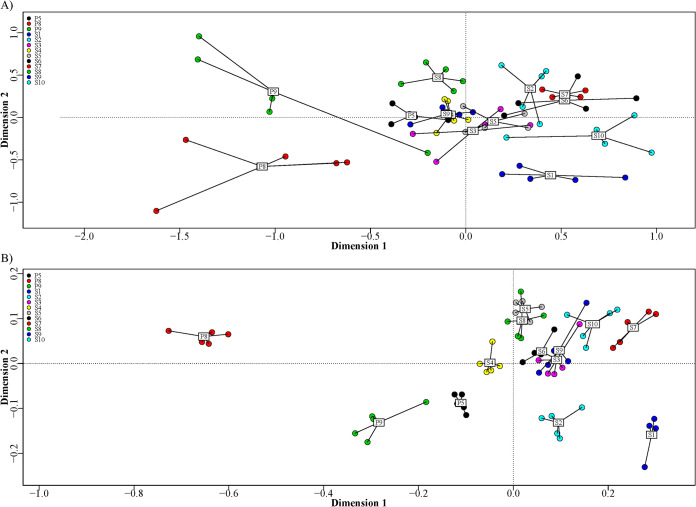
Multivariate analysis by NMDS using Bray-Curtis distances of 13 soil sample sites using amplicon sequencing of (A) the *rpoD* gene (stress 0.1883) and (B) the V3-V4 region of the rRNA gene (stress 0.1039). S1, corn; S2, fallow (grass); S3, S7, and S9, wheat; S4, rye; S5, barley; S6, rapeseed; S8, grass seed; S10, Lucerne; P5 and P9, pristine short grass; P8, pristine long grass.

Using the relative abundances (Fig. 2), the performance of the method on the natural samples was investigated across biological replicates. The most abundant species represented the four groups P. syringae, *P. lutea* (*P. graminis*), P. putida (*P. coleopterorum*), and P. fluorescens (marked by stars in Fig. 2). Within the group P. fluorescens, the five subgroups *P. jessenii* (*P. jessenii*, *P. moorei*, and *P. umsongensis*), *P. gessardii* (*P. gessardii* and *P. proteolytica*), *P. koreensis* (*P. baetica*, *P. helmanticentis*, and *P. moraviensis*), *P. mandelii* (*P. frederiksbergensis*, *P. lini*, and *P. migulae*), and *P. corrugata* (P. brassicacearum, and *P. kilonensis*) were identified. Overall, similar abundances were found in replicate samples. This was confirmed by non-metric multidimensional scaling (NMDS) analysis (Fig. 3), in which biological replicates clustered together, although with different degrees of variation. The beta dispersions of the sites were negatively correlated (*r* = −0.455) to estimated Pseudomonas load through quantitative PCR (qPCR), suggesting that the variation increased as Pseudomonas abundance decreased. The two sample sites P8 and P9 had the highest internal variation, likely owing to the low read count in these samples.

The individual sites in the *rpoD* amplicon analysis differed in species composition, despite being sampled from similar ecological environments (Fig. 2). For instance, the wheat soil samples S3, S7, and S9 had different Pseudomonas composition. *P. moraviensis*, *P. lini*, *P. helmanticensis*, and *P. frederiksbergensis* were common in most or all sampled soils; however, their relative abundances varied between sites.

**FIG 4:**
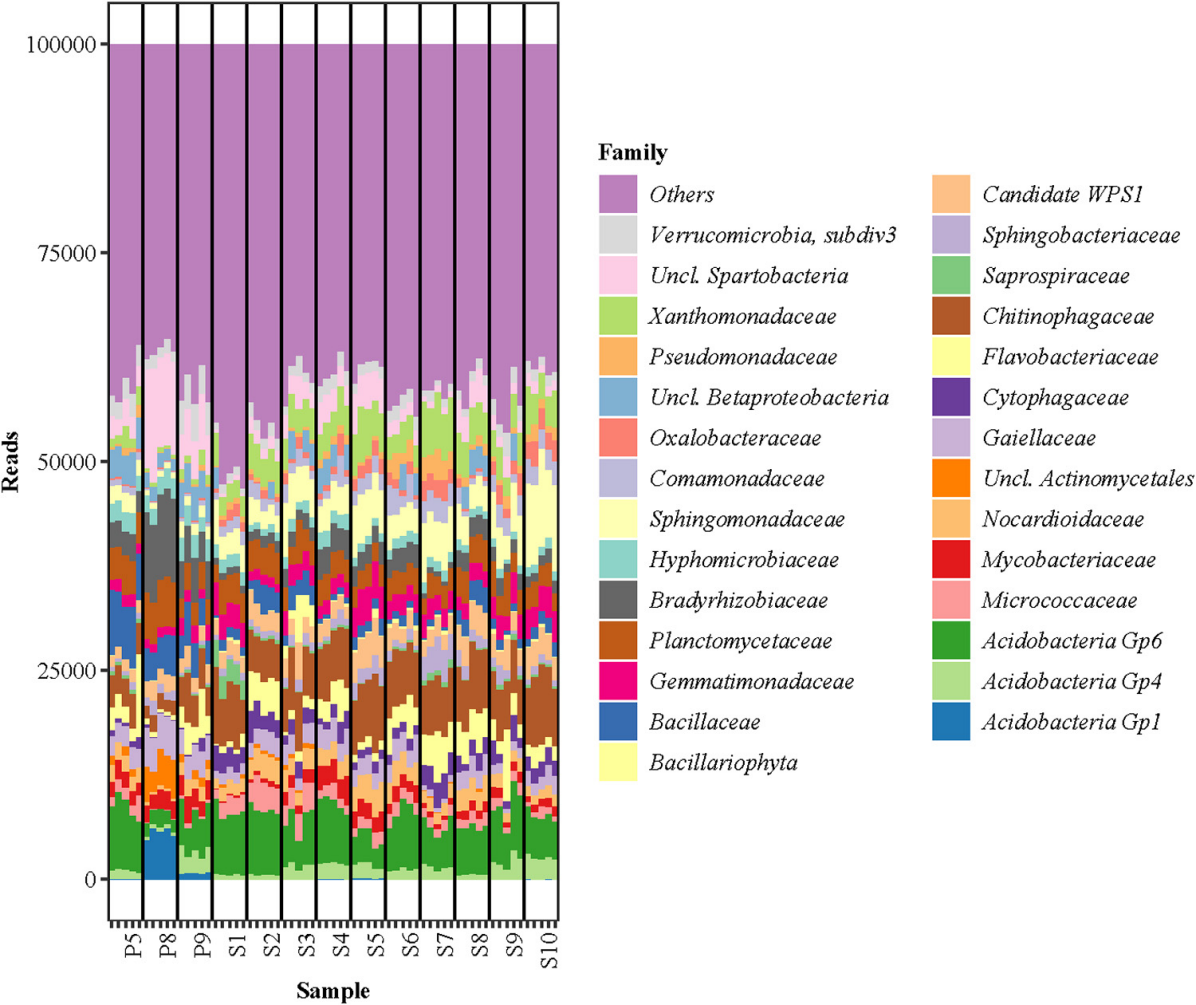
Relative abundances of the 20 most abundant bacterial families in 13 soil samples as analyzed by amplicon sequencing of the V3-V4 region of the rRNA gene. Each sample has been normalized to 100,000 reads. S1, corn; S2, fallow (grass); S3, S7, and S9, wheat; S4, rye; S5, barley; S6, rapeseed; S8, grass seed; S10, Lucerne; P5 and P9, pristine short grass; P8, pristine long grass.

The standard V3-V4 amplicon methodology resulted in a total of 6,947,525 mapped reads with an average of 106,885 per sample. A total of 378 families were identified, with the dominant families being *Xanthomonadaceae*, *Sphingomonadaceae*, *Planctomycetaceae*, *Chitinophagaceae*, and *Acidobacteria* (group 6) (Fig. 4). At the family level, only smaller variations within sample sites were observed, as visually evident in the NMDS (Fig. 3), such as the unique presence of *Acidobacteria* (group 1) in the P8 and P9 sites. The average relative abundances of Pseudomonas varied from 0.008% (P8) to 0.73% (S8) in the different communities (Table 3). At the species level, V3-V4 typically only identified one or two species in higher relative abundances, and similar to the synthetic communities, these species were assigned to be P. fluorescens and P. aeruginosa. Each site had, on average, a 70% (*P* = 0.00004) larger beta dispersion when profiled for Pseudomonas with the V3-V4 method compared to the *rpoD* method, and all had at least one sample with a species not found in the other replicates.

**TABLE 3:** Average total CFU per site estimated by qPCR and estimated relative and absolute abundance of Pseudomonas species based on V3-V4 amplicon sequencing

Site	Total bacteria (CFU/g soil)	Relative level of Pseudomonas (%)	Total Pseudomonas (CFU/g soil)
P5	1.10 × 10^8^	0.381	4.19 × 10^5^
P8	8.13 × 10^7^	0.008	6.50 × 10^3^
P9	5.30 × 10^7^	0.027	1.43 × 10^4^
S1	1.10 × 10^8^	0.288	3.17 × 10^5^
S2	3.37 × 10^7^	0.053	1.79 × 10^4^
S3	3.56 × 10^7^	0.021	7.48 × 10^3^
S4	4.81 × 10^7^	0.290	1.39 × 10^5^
S5	2.21 × 10^7^	0.065	1.44 × 10^4^
S6	2.78 × 10^7^	0.649	1.80 × 10^5^
S7	2.13 × 10^7^	0.209	4.45 × 10^4^
S8	4.29 × 10^7^	0.731	3.14 × 10^5^
S9	4.27 × 10^7^	0.126	5.38 × 10^4^
S10	2.73 × 10^7^	0.276	7.53 × 10^4^

A standard curve comparing threshold cycle (*C_T_*) values from qPCR and cell numbers was generated using pure cultures of *P. moorei* and Bacillus subtilis and used to estimate total rather than relative abundance. The average total CFU/g soil per sample site (Table 3) ranged from 2.1 × 10^7^ (S7) to 1.1 × 10^8^ (S1 and P5). The average number of Pseudomonas CFU/g soil was calculated by multiplication by the relative abundance of Pseudomonas found in the V3-V4 amplicons (Table 3) and ranged from 6.5 × 10^3^ (P8) to 4.2 × 10^5^ (P5) CFU/g soil.

### Isolation and identification of presumptive Pseudomonas.

**FIG 5:**
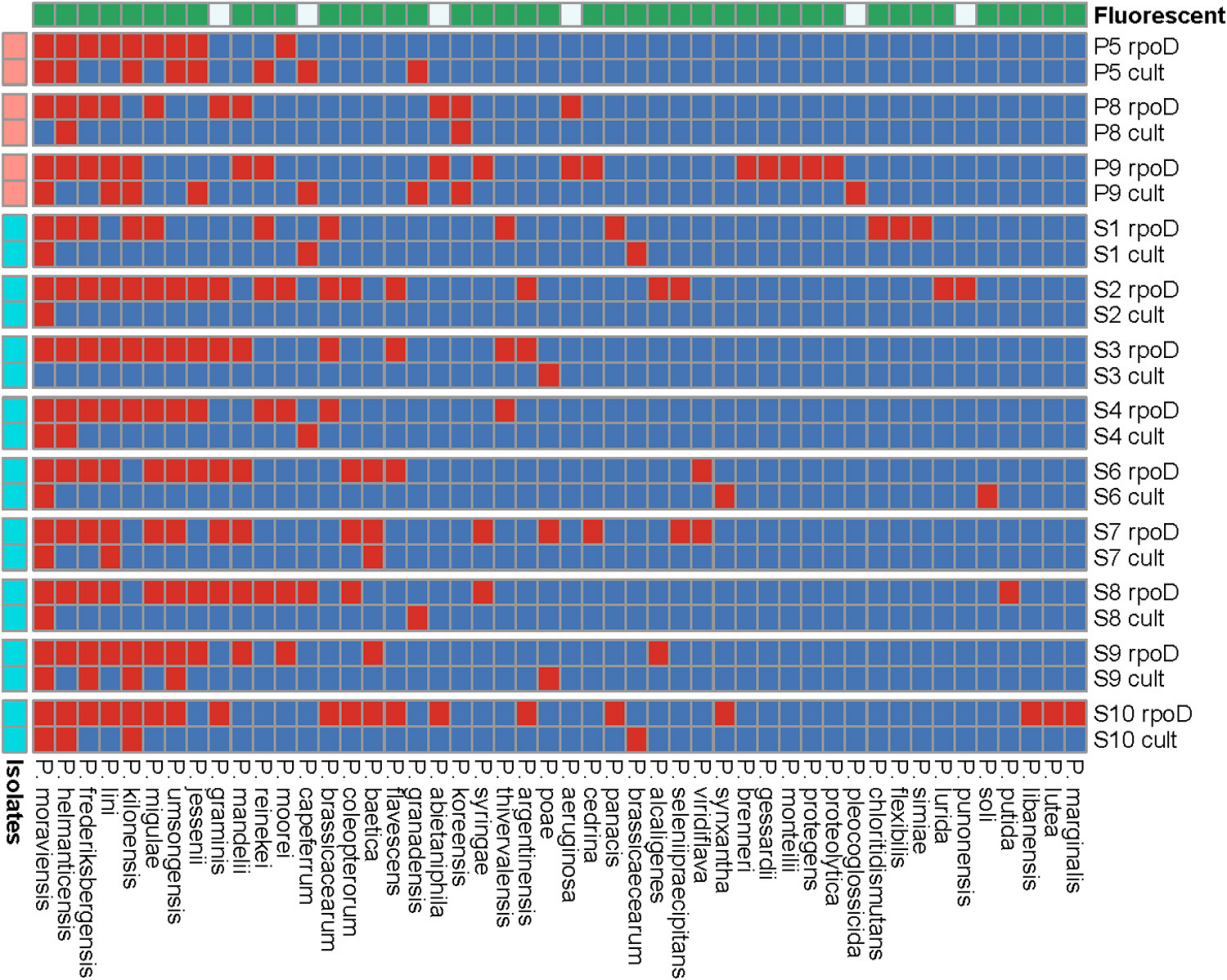
Heat map of the presence (red) and absence (blue) of Pseudomonas species identified across sites by the *rpoD* amplicon method and the cultivation approach for each of the sampled sites. Thirty isolates were sampled from each of the sites P5, P8, and P9 and 10 from the sites S1 to S10 (leftmost color annotation). Fluorescent species are highlighted by green in the upper color annotation. The row labels “rpoD” and “cult” denote analysis by *rpoD* sequencing and culture, respectively.

The *rpoD* amplicon culture-independent method for Pseudomonas species profiling was compared to cultivation-based profiling. Colonies were isolated from each of the sites on King’s agar B^+++^, which is commonly used for Pseudomonas isolation (25): 10 from each of the S1 to S10 sites and 30 for the grassland samples P5, P8, and P9. The isolates were taxonomically classified by Sanger sequencing of part of the *rpoD* gene amplified with primers PsEG30F and PsEG790R and alignment to the *rpoD* database used for the amplicon analysis. In the grassland samples, 88 of the 90 isolates were classified at the species level. Of particular interest were the species belonging to the P. fluorescens group, which encompassed 94% (83/88) of the isolates. This agrees with culture-independent profiling of P5 and P8, in which 97% (P5) and 93% (P8) of the *rpoD* reads were assigned to species in the P. fluorescens group. In P9, the fraction of species in the P. fluorescens group was lower (52%), which was predominantly due to a high fraction of *P. abietaniphila* species (39.5%), which was not observed by cultivation (Fig. 5; see Fig. S4 in the supplemental material).

10.1128/mSystems.00704-21.4 FIG S4 Number and identity of Pseudomonas species isolated by cultivation from the 13 soil sample sites. Download FIG S4, PDF file, 0.3 MB. Copyright © 2021 Lauritsen et al. 2021 Lauritsen et al. https://creativecommons.org/licenses/by/4.0/ This content is distributed under the terms of the Creative Commons Attribution 4.0 International license.

The *rpoD* amplicon method identified more unique species than the cultivation method (Fig. 5), and species belonging to particular groups (i.e., the *P. lutea* group) and subgroups (i.e., the *P. mandelii* and *P. gessardii* subgroups of P. fluorescens) were almost exclusively identified by the amplicon method but not by the cultivation-based method. The same pattern emerged in the lesser-studied sites, S1 to S10, where *rpoD* profiling identified between 12 and 19 species compared to the cultivation approach, which found between 0 and 5.

## DISCUSSION

Global food demand is growing, and since petrochemical-based industrial farming is unlikely to be sustainable for future generations, there is an urgent need for novel and sustainable biocontrol agents. Species of the Pseudomonas genus are promising as plant biocontrol agents and since beneficial traits are typically linked to particular species, we developed a high-throughput method for metataxonomic assignment of these species in natural microbiomes. The method correctly identified all species of a Pseudomonas mock community. In soil samples, *rpoD* amplicon sequencing allowed a much higher degree of Pseudomonas species differentiation than both traditional 16S rRNA V3-V4 sequencing and culturing. The *rpoD* profiling enables quick identification and prioritization of soils with specific Pseudomonas communities for further analysis and culturing.

A total of 14 primer pairs targeting the genus Pseudomonas (26–31) were examined using *in silico* PCR. The primer pair PsEG30F and PsEG790R (21) outperformed all other pairs, and in further analysis of the *rpoD* genes from 465 Pseudomonas species, two alternative forward primers were identified. However, the PsEG30F and PsEG790R primers had superior performance and were ultimately selected for further testing. Multiple studies (20, 21, 26, 32, 33) have shown that the *rpoD* gene is a good candidate for identification at the species and strain levels for the genus of Pseudomonas, especially compared to the 16S rRNA gene (20). The second-best candidate was identified as the *gap* primers of Sarkar and Guttman (28). However, these primers also amplified non-Pseudomonas species *in vitro* (data not shown) and were therefore not further considered. The PsEG30F and PsEG790R amplicons were adapted to an Illumina system, which unfortunately has too short of a read length to overlap, necessitating a new bioinformatic pipeline drawing inspiration from annotation of transcriptome sequencing (RNA-seq) data, as well as a database based on the genomes from Hesse et al. (15).

When testing the method on a known mixture of pseudomonads, *P. libanensis* and P. putida were underestimated. Through *in silico* investigation, *P. libanensis* was poorly targeted by the primers, implying poor amplification efficiency (data not shown). The *rpoD* genes of P. putida KT2440 (in the mixture), P. putida NBRC14164 (in the database), and *P. monteilii* DSM14164 (in the database) were compared in a phylogenetic tree (see Fig. S5 in the supplemental material), and although the three species are extremely closely related, KT2440 and DSM14164 are nearly identical and likely to overlap in the investigation. In the future, the *rpoD* database could be expanded to contain more strains for each species to give a wider coverage for such fringe cases. Such an addition could also lead to a strain-level differentiation in future studies. Alternatively, this occurrence could also indicate that P. putida and *P. monteilii* generally are very closely related and difficult to separate. According to Hesse et al. (15), the two species are also extremely closely related based on the protein phylogeny of 100 gene orthologues.

10.1128/mSystems.00704-21.5 FIG S5 Phylogenetic tree of the *in silico* PCR amplicons generated from the *rpoD* genes of P. putida KT2440 and four closely related species. The PCR products were aligned with MUSCLE v3.8.1551 (40), and a phylogenetic tree was calculated with FastTree v2.1.10 (41) from the alignment. Download FIG S5, PDF file, 0.04 MB. Copyright © 2021 Lauritsen et al. 2021 Lauritsen et al. https://creativecommons.org/licenses/by/4.0/ This content is distributed under the terms of the Creative Commons Attribution 4.0 International license.

The V3-V4 amplicon data for the *in vitro* DNA sample predominantly identified one species in the sample—*P. extremorientalis* or P. fluorescens—both of which belong to the subgroup P. fluorescens (15). This was also seen in the study by Mulet et al. (20), where species of the Pseudomonas genus at most had 2% dissimilarity in the 16S rRNA gene, and hence this gene does not allow a species-level resolution.

The *rpoD* amplicon methodology was used to profile the Pseudomonas population in soil samples. The relative abundances across replicates were very similar, yet some variation was observed, which could be caused by spatial differences within the soil sampled (34). Replicate variance was associated with low Pseudomonas load, as a negative correlation (*r* = −0.455) was observed between the multivariate variance of the replicates (e.g., the beta dispersion) and the observed Pseudomonas CFU/g soil. To our knowledge the closest non-16S rRNA gene-based amplicon study of Pseudomonas is by Sánchez et al. (22), where an *rpoD* amplicon methodology was used to identify Pseudomonas species in a water sample. However, a direct comparison is difficult, since Sánchez et al. (22) analyzed a single river sample with no replicates and used 454 sequencing and a blastn similarity search followed by OTU clustering rather than Illumina sequencing and bowtie mapping as in our study. The use of the 454 system, discontinued in 2016, results in longer (300 to 600 bp) single-end reads, thus avoiding the issues with nonoverlapping reads, although they are also too short to cover the entire amplicon. Use of single-end reads allows for analytically simpler OTU-based pipelines, but discards the phylogenetically import paired-end information of our approach, and hence has lower sensitivity. Sánchez et al. (22) assigned 10.8% (716 sequences) of the *rpoD* gene sequences to one of 26 species in their database. Using the genomes of Hesse et al. (15), the database now includes 166 Pseudomonas species, three of which are subspecies. Many of the *rpoD* reads were not mapped to our Pseudomonas database, which is likely due to the high stringency in our alignment approach and artifacts stemming from the low-template PCR: e.g., when Pseudomonas is in low abundance compared to other bacteria. Optimization of the PCR protocol may alleviate this.

The microbial composition as determined by V3-V4 16S rRNA amplicon sequencing was highly similar across biological replicates, with small differences between the sample sites. The three wheat-associated sites (S3, S7, and S9) clustered together, but had less overlap than a cluster with sites S3, S6, and S9, which all had different vegetation. Microbial communities in agricultural soils are influenced by physiochemical properties of the soil, the growth condition of the crops, the individual plant genotype, and/or the evolution of the microbial communities over a multitude of seasons, and the present study did not have access to such metadata that potentially could explain differences. The major outlier of the sites was the P8 site, mainly due to the high content of *Acidobacteria* (group 1), suggested to correlate with Cu and Mn concentrations (35), coinciding with a high relative abundance of *P. frederiksbergensis*.

The resolution at the species level was compared between the *rpoD* and the V3-V4 amplicon sequencing methods. The Pseudomonas species resolution based on the latter was lower than that found by the *rpoD* amplicon sequencing, which was consistent with the control experiment using 16 known species of pseudomonads. This is most likely a combination of the low dissimilarity in the 16S rRNA gene, the annotation method, and the database. It is important to note the usefulness of V3-V4 rRNA gene amplicon sequencing as a tool to determine the overall composition of the prokaryotic community. Of note, we observed different microbial communities across the different soils, even in soils having the same plant host. The *rpoD* amplicon methodology did not achieve a community-level resolution and is therefore best used in combination with standard 16S profiling to achieve full profiling of soil.

The *rpoD* profiling was compared to cultivation of Pseudomonas species from three soil samples and provided the same groups or subgroups; however, the *rpoD* amplicon sequencing method was able to identify more unique species than were found by cultivation. In particular, species belonging to the *P. lutea* group and the *P. mandelii* and *P. gessardii* subgroups of P. fluorescens were captured by the *rpoD* amplicon method but not by cultivation. The nutrient content of the isolation medium can influence the recovery of the Pseudomonas diversity from environmental samples (19). King´s B agar medium (36) is a nutrient-rich medium for Pseudomonas isolation, and while this is an effective and commonly used cultivation method, it is possible that a larger number of Pseudomonas species could have been cultivated using a wider range of cultivation media. Overall, the *rpoD* amplicon methods can be used to find soil rich in Pseudomonas species and identify samples rich in potential beneficial or useful pseudomonads.

A few species were exclusively found by the culture-independent approach, and some of these are promising as bioinoculants for plant protection (Fig. 5). For example, strains of Pseudomonas frederiksbergensis (from the *P. mandelii* subgroup), which were found in all *rpoD* profiles, but only once by cultivation (in sample S9), are effective bioinoculants for enhancing cold stress tolerance in plants (37). In addition, Pseudomonas abietaniphila (from the *P. lutea* group), which was found in three *rpoD* profiles (P8, P9, and S10) but not in any cultivations, can suppress plant diseases caused by Botrytis cinerea by degradation of oxalate produced by the fungi (38). Also, we have recently shown that the genes for biosynthesis of the antifungal compound thioquinolobactin are rarely found, but are tightly linked to biocontrol strains within the *P. gessardii* subgroup of P. fluorescens (39). Here, we find such species in P9 only with the cultivation-independent method.

With the *rpoD* amplicon approach, it is possible to profile and prioritize samples for intensive cultivation of strains that produce specific bioactive metabolites for biocontrol or exhibit other plant beneficial traits.

### Conclusion.

In this study, an *rpoD* gene amplicon-based technique to differentiate species within the genus Pseudomonas was developed. The method can differentiate individual species far beyond what traditional 16S rRNA gene amplicon sequencing can and is proposed as a new standard for high-throughput profiling of Pseudomonas in environmental microbial communities.

## MATERIALS AND METHODS

### *In silico* investigations of Pseudomonas species.

A Pseudomonas genome collection consisting of the 165 genomes from Hesse et al. (15) as well as 465 complete genome sequences of Pseudomonas was downloaded from NCBI (downloaded 18 February 2019). Using an *in silico* PCR algorithm (*in_silico_*PCR; https://github.com/egonozer/in_silico_pcr) (one mismatch, one deletion/insertion), 14 primer sets targeting nine different genes from previous studies (Table 1) were evaluated based on (i) the proportion of Pseudomonas genomes amplified, (ii) how well the amplicons followed their given phylogenetic classification, and (iii) the proportion of non-Pseudomonas genomes amplified (see Table S1 in the supplemental material). The *in silico* PCR products were aligned with MUSCLE v3.8.1551 (40), and a phylogenetic tree was generated with FastTree v2.1.10 (41) from the alignment. For phylogenetic evaluation, the output tree was qualitatively compared to the whole-genome-based tree of Hesse et al. (15). A custom database was built by using the PsEG30F/PsEG790R primers on the *rpoD* genes of the type strains included in the study by Hesse et al. (15). An issue encountered in the *in silico* analysis was the poor annotations of uploaded genomes, where we found multiple instances of incorrectly annotated genomes, which we corrected by selecting the *rpoD* genes of outliers and reidentifying them according to the type strain genomes of Hesse et al. (15).

10.1128/mSystems.00704-21.6 TABLE S1 Genomes of non-Pseudomonas species used to test Pseudomonas gene primer specificity profiling. Download Table S1, DOCX file, 0.01 MB. Copyright © 2021 Lauritsen et al. 2021 Lauritsen et al. https://creativecommons.org/licenses/by/4.0/ This content is distributed under the terms of the Creative Commons Attribution 4.0 International license.

### Pseudomonas strains.

Sixteen different Pseudomonas species were used in the *in vitro* testing of the *rpoD* amplicon (Table 2). Seven were type culture collection strains, and nine were isolates obtained from ongoing projects in our laboratory. They were identified to the species level by Sanger sequencing of the *rpoD* gene as described above. The strains were grown in 10 ml Luria-Bertani (LB) broth (Lennox; Carl Roth GmbH & Co. KG, Karlsruhe, Germany) overnight at 30°C with aeration (shaking, 200 rpm).

### Soil samples.

Bulk soil samples were collected from 13 sites in mid-August 2019 close to harvest season. The samples were collected by scooping root-associated soil into a sterile Falcon tube. The sites were distributed across Zealand, Denmark, and included different types of vegetation and produce; 10 samples of field soil were collected, including corn (S1), fallow (grass [S2]), wheat (S3, S7, and S9), rye (S4), barley (S5), rapeseed (S6), grass seed (S8), and Lucerne (S10). In addition, three samples of pristine grassland were collected, including short (P5 and P9) and tall (P8) grass. The soil samples were stored at 5°C for a maximum of 2 weeks prior to analyses.

### Isolation of Pseudomonas from soil samples.

Soil was sieved (4.75- by 4.75-mm grid) and mixed with 0.9% NaCl in a ratio of 1 g to 9 ml and 10-fold serially diluted. Dilutions were plated on 1/4-diluted King’s B^+++^ agar plates (Sigma-Aldrich) supplemented with 13 mg/liter chloramphenicol, 40 mg/liter ampicillin, and 100 mg/liter cycloheximide (25). The plates were incubated at 30°C for 5 days. The plates were examined under UV light after 2 and 5 days to reveal fluorescent colonies. LB agar plates were streaked with up to 30 colonies from the P5, P8, and P9 sites as well as 10 from the S1 to S10 sites and incubated at 30°C for 2 days. The colonies were selected based on fluorescence and distinct colony morphology.

### DNA extraction from pure cultured Pseudomonas and soil samples.

For identification of Pseudomonas isolated from soil, DNA from each bacterial colony was extracted by boiling in demineralized H_2_O (dH_2_O) at 99°C for 15 min. For soil samples and selected Pseudomonas strains, genomic DNA (gDNA) was extracted with a DNAeasy Powersoil kit (Qiagen, Hilden, Germany) according to the manufacturer's instructions. The extractions of gDNA from soil were done in five biological replicates for each site. As a negative control, 250 μl sterile dH_2_O was extracted for gDNA using the same methodology. The gDNA was stored at −20°C. The DNA extraction of soil was done at the latest 2 days after cultivation of soil Pseudomonas.

### Identification of Pseudomonas isolates from soil samples.

Pseudomonas species isolated from soil were identified by full-length sequencing of the *rpoD* gene. The 25-μl PCR mixture contained 10.6 μl Sigma Water, 12.5 μl 2× TEMPase, 0.8 μl forward primer (10 μM PsEG30F [5′-ATYGAAATCGCCAARCG-3′]), 0.8 μl reverse primer (10 μM PsEG790R [5′-CGGTTGATKTCCTTGA-3′]), and 0.3 μl template DNA. The PCR program was (i) 1 cycle of 95°C for 15 min, (ii) 30 cycles of 95°C for 30 s, 51°C for 30 s, and 72°C for 30 s, and (iii) 1 cycle of 72°C for 5 min (21). PCR products were sequenced at Macrogen Europe (Amsterdam, The Netherlands). The *rpoD* sequences were classified using blastn against the custom-built *rpoD* gene database.

### Defined *in vitro* DNA mixture for Pseudomonas profiling.

As a positive control, a defined Pseudomonas DNA mixture was made as an equimolar mixture of individual extractions of the strains in Table 2, as measured by Nanodrop (Denovix DS-11; Saveen & Werner AB, Linhansvãgen, Sweden). The equimolar mixture was based on DNA concentrations.

### Amplicon preparation, purification, and sequencing.

Amplicons were prepared by amplifying DNA using barcoded primers (see Table S2 in the supplemental material). The five biological replicates of each soil site, five technical replicates of the *in vitro* DNA mixture (positive control), and the negative control all were amplified using both the *rpoD*-specific primers and primers targeting the V3-V4 region of the 16S rRNA gene. Each sample used identical barcodes across both primer sets (Table S2) and Illumina adaptors for the two setups. For the amplification of *rpoD* genes, a 25-μl PCR mixture containing 10.15 μl Sigma Water, 12.5 μl 2× TEMPase, 0.8 μl forward primer (10 μM barcoded PsEG30), 0.8 μl reverse primer (10 μM barcoded PsEG790), 0.25 μl MgCl_2_ (25 mM), and 0.5 μl template DNA was used. The PCR program was as follows: (i) 15 min at 95°C, (ii) 40 cycles of 30 s at 95°C, 30 s at 51°C, and 30 s at 72°C, and (iii) 5 min at 72°C. The amplicons were stored at −20°C until purification.

10.1128/mSystems.00704-21.7 TABLE S2 List of barcodes tagged on primers for Illumina sequencing of amplicons of the Pseudomonas *rpoD* gene. Download Table S2, DOCX file, 0.01 MB. Copyright © 2021 Lauritsen et al. 2021 Lauritsen et al. https://creativecommons.org/licenses/by/4.0/ This content is distributed under the terms of the Creative Commons Attribution 4.0 International license.

For the amplification of V3-V4 regions, a 25-μl PCR mixture containing 10.6 μl Sigma Water, 12.5 μl 2× TEMPase, 0.8 μl forward primer (10 μM barcoded 341F [5′-CCTACGGGNGGCWGCAG-3′]), 0.8 μl reverse primer (10 μM barcoded 805R [5′-GACTACHVGGGTATCTAATCC-3′]), and 0.3 μl template DNA was used (42). The PCR program was as follows: (i) 15 min at 95°C, (ii) 30 cycles of 30 s at 95°C, 30 s at 60°C, and 30 s at 72°C, and (iii) 5 min at 72°C. The amplicons were stored at −20°C until purification.

The amplicons were purified using an Agencourt AMPure XP kit (Beckman Coulter, Brea, CA, USA) following the manufacturer's instructions. The products were eluted in Tris (10 mM, pH 8.5) buffer. After purification, the PCR products were pooled in equimolar concentrations.

The amplicon pools were delivered to the CfB NGS Lab (Novo Nordisk Foundation Center for Biosustainability, DTU, Kongens Lyngby, Denmark) for sequencing on an Illumina MiSeq 300PE platform (MiSeq reagent kit v3; PE300).

### Enumeration of soil bacteria.

The number of cells in each soil site was quantified using quantitative PCR (qPCR). The qPCR targeted the V3 region of the 16S rRNA gene using the primers 338F and 518R (43). A 20-μl PCR mixture containing 5.2 μl Sigma Water, 10 μl Luna Universal qPCR master mix (New England Biolabs, Inc., Bionordika Denmark A/S, Denmark), 1.4 μl of each primer (10 μM), and 2 μl template DNA was used. The accompanying instructions for the qPCR program were followed. A standard curve relating cycle thresholds (*C_T_*) to CFU/g soil was prepared by combining CFU/g versus *C_T_* for Bacillus subtilis ATCC 6051 and Pseudomonas moorei DSM 12647 (*R*^2^ = 0.86, E = 174.5%). ATCC 6051 and DSM 12647 were incubated overnight in 5 ml LB broth at 30°C with aeration. At an optical density at 600 nm (OD_600_) of approximately 1 (circa 24 h of growth), DNA was extracted from the cultures and further diluted. The standard curves were prepared in biological duplicates.

### Processing the V3-V4 and *rpoD* amplicons.

The V3-V4 amplicons were cleaned, merged, quality filtered, and chimera checked before quality-aware clustering at 99% similarity and mapping against the RDP-II SSU database (v11.5) (46) using the BION-meta software (Danish Genome Institute, Aarhus, Denmark). For the *rpoD* amplicons (PsEG30F and PsEG790R), the BION-meta software (Danish Genome Institute, Aarhus, Denmark) was used to demultiplex the amplicons. The fastp function (44) was used for quality filtering. Since the paired reads do not overlap, clustering was avoided, and instead, each read pair was aligned to the custom database of *rpoD* genes using bowtie2 (23). The resulting SAM-file was then filtered for only concordant pairs mapped with a quality of >10 using samtools (45). Data for both sets of amplicons were normalized to 100,000 reads for each sample before further analysis. Centrifuge was used for profiling of non-Pseudomonas reads using the *p+h*+v database (24).

### Statistics.

The amplicon sequencing data for both *rpoD* and V3-V4 were analyzed by non-metric multidimensional scaling (NMDS) to compare the diversities between the replicates and sample sites. To determine the multivariable variation within groups, the beta dispersion was calculated using R v3.6.2 package vegan with default settings and tested using a Mann-Whitney U test. Multiple distances were evaluated for robustness, and the Bray-Curtis distance was chosen since this distance metric had the best trade-off in terms of separation of sites and stress of the NMDS.

### Data availability.

The raw amplicon sequencing data from the Illumina sequencing are available at the Sequencing Read Archive (SRA) as BioProject PRJNA613913. Code for both *in silico* primer analysis and the bioinformatic classification pipeline is available at https://github.com/mikaells/PseudomonasRPOD.
