# Supplementary figures and images for: Identification and Differentiation of Pseudomonas Species in Field Samples Using an rpoD Amplicon Sequencing Methodology
Source: mSystems. 2021 Aug 3;6(4):e00704-21. doi: 10.1128/mSystems.00704-21 (PMC8407407; doi:10.1128/mSystems.00704-21)

A)

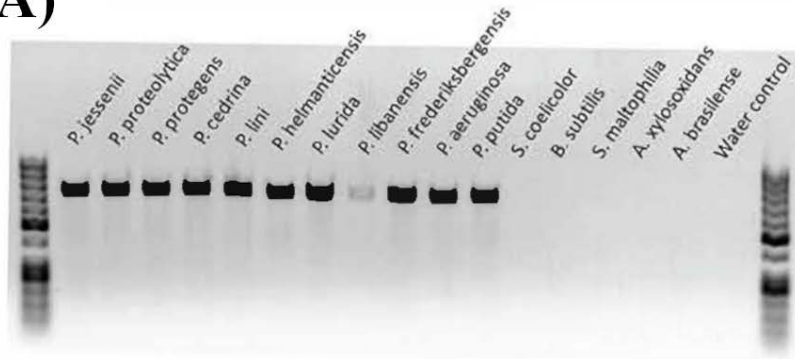

B)

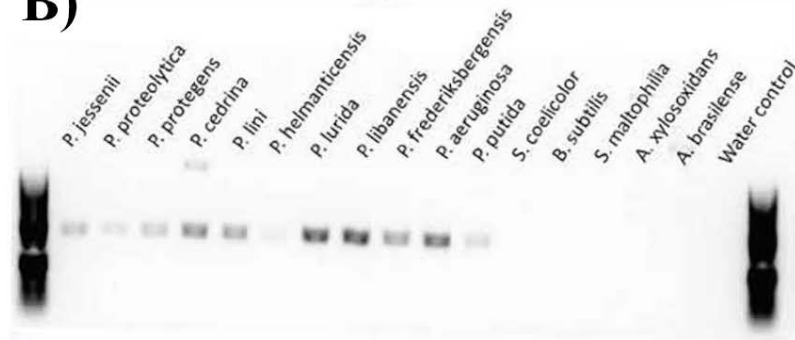

C)

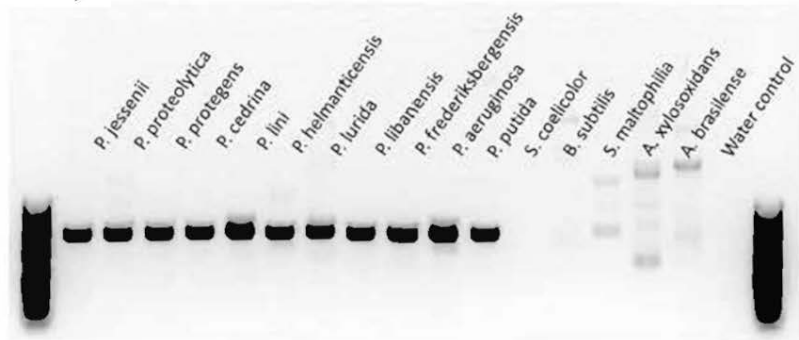

Supplement: FIG S1 [file msystems.00704-21-sf001.pdf]

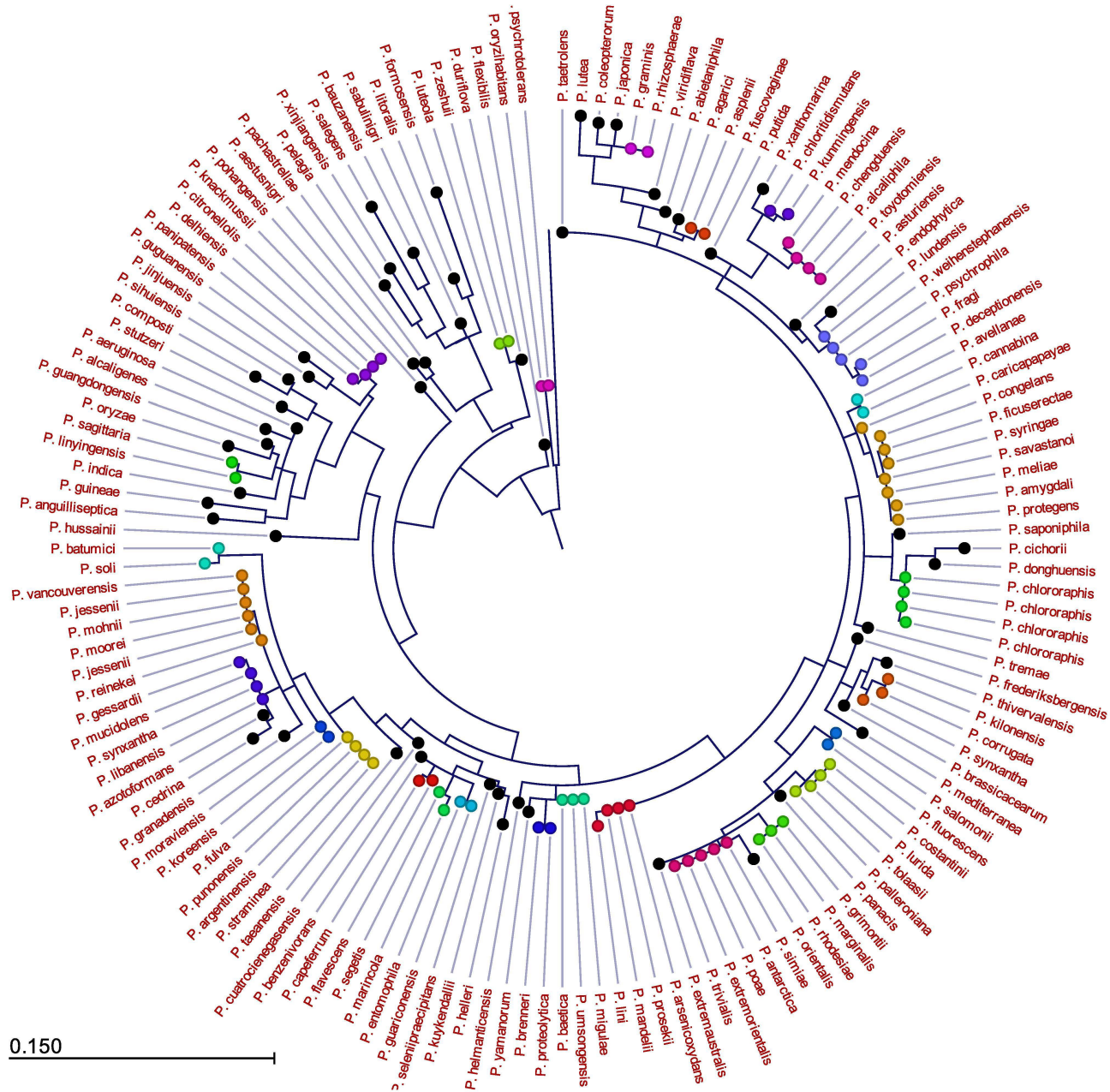

Supplement: FIG S2 [file msystems.00704-21-sf002.pdf]

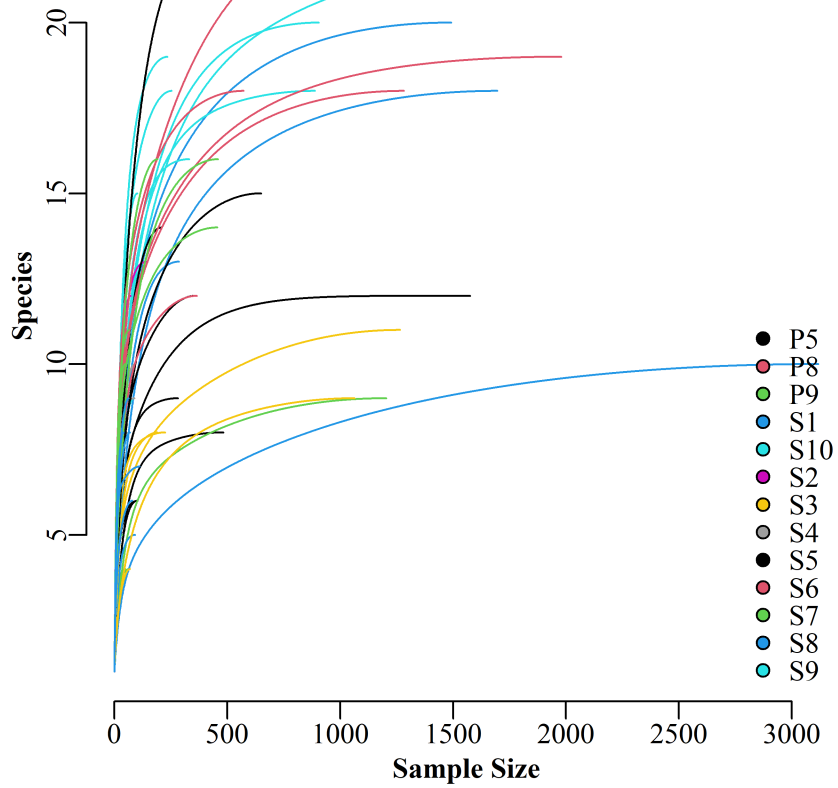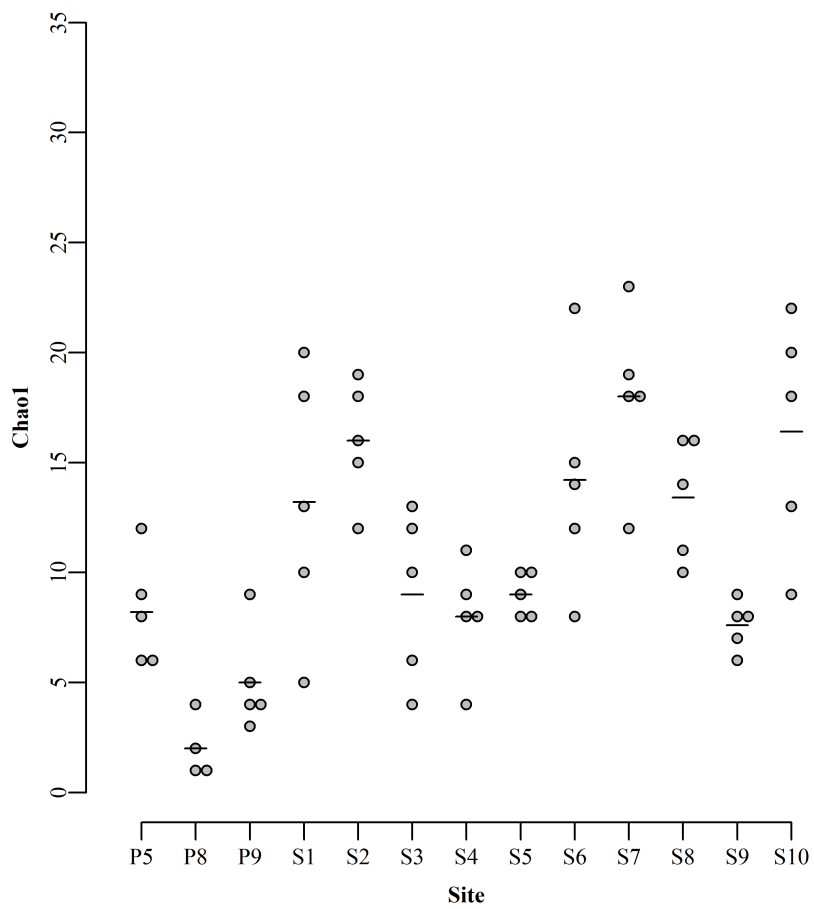

Supplement: FIG S3 [file msystems.00704-21-sf003.pdf]

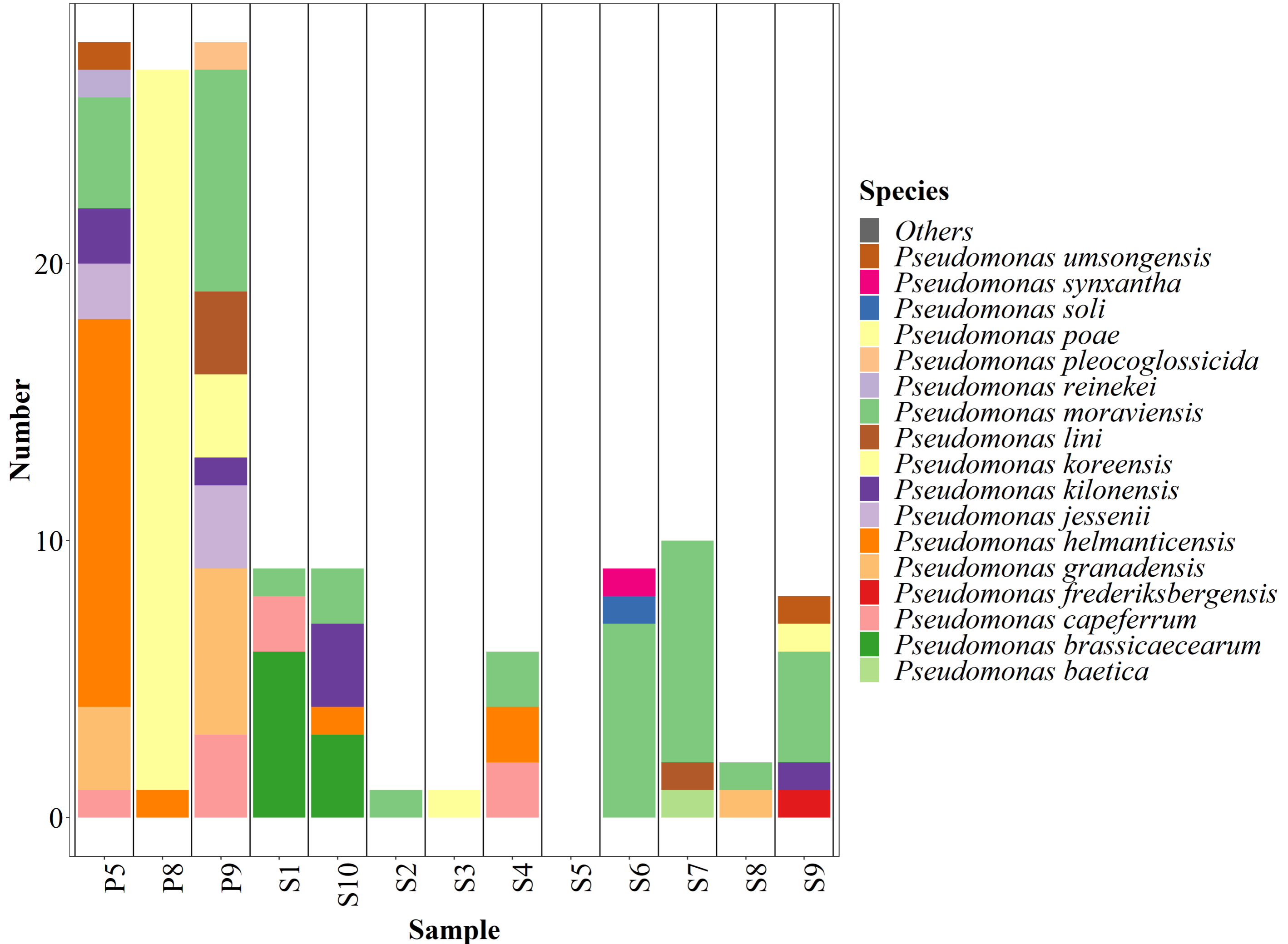

Supplement: FIG S4 [file msystems.00704-21-sf004.pdf]

# rpoD similarities between the custom database and KT2440

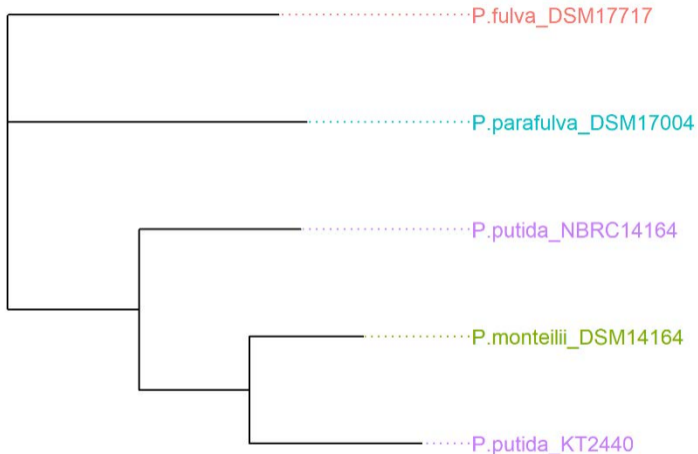

0.008

—

Supplement: FIG S5 [file msystems.00704-21-sf005.pdf]
