# Supplementary material for: Identification and Differentiation of Pseudomonas Species in Field Samples Using an rpoD Amplicon Sequencing Methodology
Source: mSystems. 2021 Aug 3;6(4):e00704-21. doi: 10.1128/mSystems.00704-21 (PMC8407407; doi:10.1128/mSystems.00704-21)
Supplement: TABLE S3 [file msystems.00704-21-st003.docx]

**Table S3**. *In silico* PCR amplification of 165 genomes derived from Hesse et al. (15), 465 complete *Pseudomonas* genomes derived from NCBI and 24 non-*Pseudomonas* genomes using 14 different primer pairs.

|  | Number of *in silico* PCR products (%) | | |
| --- | --- | --- | --- |
| Primer pair | Hesse et al^15^. *Pseudomonas* | NCBI complete *Pseudomonas* | Non-*Pseudomonas* |
| 16S-341F / 16S-805R | 146/166 (87.95) | 465/465 (100.00) | 18/24 (75.00) |
| 16sF-LYP-3 / 16sR-LYP-3 | 0/166 (0.00) | 0/465 (0.00) | 0/24 (0.00) |
| 16S-rRNA-F / 16S-rRNA-R | 132/166 (79.52) | 465/465 (100.00) | 0/24 (0.00) |
| atpD-F / atpD-F | 66/166 (39.76) | 286/465 (61.51) | 4/24 (16.67) |
| carA-F / carA-R | 70/166 (42.17) | 291/465 (62.58) | 4/24 (16.67) |
| gapA-Fps / gapA-Rps | 90/166 (54.22) | 369/465 (79.35) | 0/24 (0.00) |
| glt-F / glt-R | 14/166 (8.43) | 40/465 (8.60) | 0/24 (0.00) |
| gyrBBAUP2 / APrU | 0/166 (0.00) | 1/465 (0.22) | 0/24 (0.00) |
| UP-1E / APrU | 166/166 (100.00) | 465/465 (100.00) | 6/24 (25.00) |
| recA-F / recA-R 409 | 109/166 (65.66) | 416/465 (89.46) | 2/24 (8.33) |
| LAPS / LAPS27 | 118/166 (71.08) | 426/465 (91.61) | 0/24 (0.00) |
| PsEG30F / PsJL490R | 148/166 (89.16) | 455/465 (97.85) | 0/24 (0.00) |
| PsEG30F / PsJL628R | 139/166 (83.73) | 456/465 (98.06) | 0/24 (0.00) |
| PsEG30F / PsEG790R | 160/166 (96.39) | 460/465 (98.92) | 0/24 (0.00) |
