# Supplementary material for: Identification and Differentiation of Pseudomonas Species in Field Samples Using an rpoD Amplicon Sequencing Methodology
Source: mSystems. 2021 Aug 3;6(4):e00704-21. doi: 10.1128/mSystems.00704-21 (PMC8407407; doi:10.1128/mSystems.00704-21)
Supplement: TABLE S1 [file msystems.00704-21-st001.docx]

**Table S1**: Genomes of non-*Pseudomonas* used to test *Pseudomonas* gene primer specificity profiling.

| **Phyla/Division** | **Species** | **Strain** | **RefSeq accession** |
| --- | --- | --- | --- |
| Actinobacteria | *Mycobacterium tuberculosis* | HN-506 | GCF_002357975.1 |
| Actinobacteria | *Streptomyces coelicolor* | A3(2) | GCF_000203835.1 |
| Actinobacteria | *Bifidobacterium bifidum* | S6 | GCF_003390735.1 |
| Firmicutes | *Bacillus subtilis* | 3610 | GCF_002055965.1 |
| Firmicutes | *Clostridium acetobutylicum* | ATCC 824 | GCF_000008765.1 |
| Firmicutes | *Staphylococcus aureus* | NCTC 8325 | GCF_000013425.1 |
| Sphingobacteria | *Sphingobacterium sp.* | 21 | GCF_000192845.1 |
| Sphingobacteria | *Sphingobacterium sp.* | B29 | GCF_001952815.1 |
| Alphaproteobacteria | *Azospirillum brasilense* | Az39 | GCF_000632475.1 |
| Alphaproteobacteria | *Agrobacterium tumefaciens* | S33 | GCF_001551895.1 |
| Alphaproteobacteria | *Agrobacterium tumefaciens* | Ach5 | GCF_000971565.1 |
| Betaproteobacteria | *Achromobacter xylosoxidans* | MN001 | GCF_001051055.1 |
| Betaproteobacteria | *Bordetella pertussis* | Tohama I | GCF_000195715.1 |
| Betaproteobacteria | *Azospira oryzae* | PS | GCF_000236665.1 |
| Gammaproteobacteria | *Stenotrophomonas maltophilia* | K279a | GCF_000072485.1 |
| Gammaproteobacteria | *Pectobacterium carotovorum* | 14A | GCF_003932035.1 |
| Gammaproteobacteria | *Salmonella enterica* | SL1344 | GCF_000210855.2 |
| Gammaproteobacteria | *Xanthomonas axonopodis* | LMG26789 | GCF_003698225.1 |
| Ascomycota | *Botrytis cinerea* | B05.10 | GCF_000143535.2 |
| Ascomycota | *Aspergillus niger* | CBS 513.88 | GCF_000002855.3 |
| Ascomycota | *Fusarium oxysporum* | 4287 | GCF_000149955.1 |
| Ascomycota | *Penicillium expansum* | MD-8 | GCF_000769745.1 |
| Ascomycota | *Saccharomyces cerevisiae* | S288C | GCF_000146045.2 |
| Nematoda | *Caenorhabditis elegans* | Bristol N2 | GCF_000002985.6 |
